# Extraintestinal Infections Caused by Non-toxigenic *Vibrio cholerae* non-O1/non-O139

**DOI:** 10.3389/fmicb.2016.00144

**Published:** 2016-02-11

**Authors:** Goutam Chowdhury, Sangeeta Joshi, Sanjay Bhattacharya, Uma Sekar, Balaji Birajdar, Arpita Bhattacharyya, Sumio Shinoda, Thandavarayan Ramamurthy

**Affiliations:** ^1^Department of Bacteriology, National Institute of Cholera and Enteric DiseasesKolkata, India; ^2^Manipal HospitalBangalore, India; ^3^Tata Medical CenterKolkata, India; ^4^Sri Ramachandra Medical CentreChennai, India; ^5^Metropolis Healthcare Ltd-Global HospitalMumbai, India; ^6^Collaborative Research Centre of Okayama University for Infectious Diseases in India, National Institute of Cholera and Enteric DiseasesKolkata, India; ^7^Translational Health Science and Technology Institute, NCR Biotech Science ClusterFaridabad, India

**Keywords:** *V. cholerae*, UTI, hematuria, septicaemia, *ompW-*PCR, serogroup identification

## Abstract

*Vibrio cholerae* is an aerobic, sucrose fermentative Gram-negative bacterium that generally prevails in the environment. Pathogenic *V. cholerae* is well-known as causative agent of acute diarrhea. Apart from enteric infections, *V. cholerae* may also cause other diseases. However, their role in causing extraintestinal infections is not fully known as it needs proper identification and evaluation. Four cases of extraintestinal infections due to *V. cholerae* non-O1/non-O139 have been investigated. The isolates were screened for phenotypic and genetic characteristics with reference to their major virulence genes. Serologically distinct isolates harbored *rtx, msh*, and *hly* but lacked enteric toxin encoding genes that are generally present in toxigenic *V. cholerae*. Timely detection of this organism can prevent fatalities in hospital settings. The underlying virulence potential of *V. cholerae* needs appropriate testing and intervention.

## Introduction

The Gram-negative bacillus *Vibrio cholerae* is classified into serogroups based on the somatic “O” surface antigen. Broadly, they are divided into O1, O139, and non-O1/non-O139 serogroups (Sack et al., 2004). *V. cholerae* O1 is generally regarded as a non-invasive enteric organism associated with gastroenteritis of varying severity (Sack et al., 2004). The serogroups O1 and O139 are associated with cholera infection, whereas the non-O1/non-O139 serogroups are commonly found as an autochthonous microbe in coastal and marine environments, which may cause cholera-like diarrhea. Several major virulence factors such as cholera toxin (CT), responsible for excess fluid secretion and the toxin coregulated pilus (TCP), involved in intestinal colonization have been reported. *V. cholerae* non-O1/non-O139 serogroups generally lack CT and TCP causes infection through other virulence factors including the heat-stable toxin (NAG-ST) and hemolysin (Hly) a type III secretion system (TTSS; Dziejman et al., 2005; Bag et al., 2008) and cholix toxin (Chx; Purdy et al., 2010). Several of these virulence factors have been documented in clinical and environmental *V. cholerae* non-O1/non-O139 isolates from Italy (Ottaviani et al., 2009), Mexico (Lizárraga-Partida and Quilici, 2009), USA (Purdy et al., 2010; Ceccarelli et al., 2015), China (Luo et al., 2013), and India (Rajpara et al., 2013).

Although biochemically indistinguishable from enteric infection causing *V. cholerae* O1/O139, the non-O1/non-O139 serogroups have been associated with extraintestinal infection including septicemia, wound infection, peritonitis, skin infection, cellulitis, necrotizing fasciitis, endophthalmitis, ear infection, cholecystitis, and meningitis (CDC, 2014; Chen et al., 2015; Hao et al., 2015). Here we report one case of urinary tract infection (UTI) and three cases of septicemia associated with diverse manifestations caused by various serogroups of *V. cholerae* non-O1/non-O139.

## Case 1

A 60-years-old female came to Manipal Hospital, Bangalore for an emergency treatment with a history of three episodes of hematuria 3 days earlier. She was suffering from diabetes and hypertension for the last couple of years and was under treatment. She gave a history of UTI after a long train journey and was asymptomatic for 3 days after which she had hematuria with dysuria. She had no previous history of UTI, blood transfusion, alcohol intake or drug abuse. She also had no recent history of diarrhea or fever. Ultrasound of abdomen did not show any abnormality. At the hospital, the preliminary diagnosis was suspected UTI and urine was collected for culture and empirical ofloxacin 200 mg was prescribed. Urine analysis revealed 10–15 pus cells per high power field and there was no blood, glucose or protein. Urine culture was done on 5% sheep blood agar and MacConkey agar. After 24 h of incubation, bacterial growth of 10^5^ cfu/ml was detected on blood agar as identical β-hemolytic colonies. Three to five randomly picked colonies were tested for Gram-staining and biochemical characteristics (World Health Organization [WHO], 1987). All the tested isolates were identified as Gram-negative curved/straight rods and *V. cholerae* by biochemical tests. One of the representative isolate was sent to National Institute of Cholera and Enteric Diseases (NICED), Kolkata for further confirmation and molecular characterization. The isolate was confirmed as *V. cholerae* by using the API-32E (bioMérieux, Marcy l’Etoile, France) and by PCR targeting the *ompW* which encodes outer membrane protein-W (Nandi et al., 2000). By slide agglutination, the isolate (U13533) was identified as serogroup O2. Antibiotic susceptibility tests (CLSI, 2014) revealed that the isolate was susceptible to all the tested antibiotics (**Table 1**). The urine examination was repeated after 3 days which revealed 2–4 pus cells with no growth in culture. Oral ofloxacin 200 mg was prescribed for 5 days and the patient was asymptomatic on follow up.

**Table 1:** Antimicrobial susceptibility results of *Vibrio cholerae* non-O1/non-O139 isolates.

Antimicrobial	Case 1 U13533	Case 2 B748	Case 3 SRMC-1	Case 4 VC-3
Ampicillin	S	S	R	S
Azithromycin	S	S	S	S
Ceftriaxone	S	S	S	S
Chloramphenicol	S	S	S	S
Ciprofloxacin	S	S	S	S
Gentamicin	S	S	S	S
Imipenem	S	S	S	S
Norfloxacin	S	S	S	S
Ofloxacin	S	S	S	S
Tetracycline	S	S	S	S
Trimethoprime-sulfamethoxazole	S	S	S	S

*R, resistance; S, susceptible.*

## Case 2

A six and half-year old female child from Howrah, West Bengal, was admitted to the Tata Medical Center, Kolkata in April 2013. She had a history of abdominal pain with occasional vomiting for a month, and rapid respiration for a day. Examination revealed tachypnoea, an abdominal mass, enlarged cervical lymph nodes, and features of bilateral pleural effusion. A diagnosis of Burkitt’s lymphoma was made on the basis of flow cytometry of pleural fluid as well as marrow morphology and immunophenotype. Chemotherapy was commenced. She developed severe respiratory distress requiring ICU admission, tracheal intubation and mechanical ventilation. She suffered an episode of cardiac arrest following which she developed features of hypoxic encephalopathy with seizures, decerebrate posturing and extensor plantar reflexes. She became febrile after 3 weeks of ICU admission. Two sets of blood (from central venous line and peripheral vein) were drawn for bacterial culture before administering empirical antibiotics, namely cefepime-tazobactam and teicoplanin. Blood cultures showed uniform β-hemolytic colonies on blood agar plates. Three suspected colonies were identified as *V. cholerae* using Vitek 2 compact system (bioMérieux). The bacterial isolate (B748) was sent to NICED for confirmation. This isolate was confirmed as *V. cholerae* by *ompW* PCR and serotyped as O8. CLSI (2014) guidelines were followed for antimicrobial susceptibility testing by disk diffusion method. The isolate B748 was susceptible to all the tested antibiotics (**Table 1**). Ciprofloxacin was added to the treatment according to the sensitivities obtained. She recovered from the episode of sepsis, but an MRI scan showed extensive hypoxic cerebral necrosis. After prolonged discussions with the family, she was sent home on palliative care, and she died 6 weeks after her initial presentation.

## Case 3

A 56-years-old lady was admitted to the Sri Ramachandra Medical College, Chennai, with chills, low blood pressure, and painful swelling of the right lower leg. She was diagnosed with diabetic nephropathy with peripheral neuropathy. There was no fever, diarrhea, nausea, or vomiting reported on admission. Physical examination revealed hemorrhagic cellulitis with tenderness in her right leg, but there was no bullous skin lesion. After admission, two sets of blood samples were drawn for culture before empiric therapy with parenteral piperacillin tazobactam. Treatment with intravenous fluid and supportive therapy was initiated. By the morning after her admission, the patient’s temperature had risen, and the hemorrhagic cellulitis had rapidly progressed. Emergency fasciotomy was performed, and parenteral antibiotic therapy was subsequently changed to imipenem. However, cellulitis with bullous lesions rapidly progressed in the right leg, in size and number. After 18 h, respiratory failure developed and she became deeply confused. She received fluid resuscitation with vasopressor therapy, intubation and ventilator care for the septic condition. However, after 24 h of admission, she died despite aggressive cardiopulmonary resuscitation. Blood culture results revealed microbial growth after 24 h of incubation of sample inoculated on 5% sheep blood agar plates. The colonies evenly exhibited β-hemolysis. Five randomly selected colonies were oxidase-positive and identified as Gram-negative rods by microscopy. Upon subculture, the colonies grew on thiosulfate-citrate-bile salts-sucrose (TCBS) agar as sucrose fermenting yellow colonies. It grew in 10% NaCl and was lysine and ornithine decarboxylase positive and arginine dihydrolase negative. The isolate was sent to NICED for further confirmation and other molecular characteristics. API-32E identification (bioMérieux) and the *ompW* PCR confirmed the isolate (SRMC-1) as *V. cholerae* that belonged to serogroup O10. CLSI (2014) guidelines were followed for antimicrobial susceptibility testing. The isolate was susceptible *in vitro* to first, second and third generation cephalosporins; imipenem; gentamicin; trimethoprim-sulfamethoxazole; tetracycline and ciprofloxacin but resistant to ampicillin (**Table 1**).

## Case 4

A 72-years-old male with chills and generalized weakness for 7 days, was admitted to the Global Hospital in Parel, Mumbai on July, 2013. On the day of admission, the patient’s temperature peaked to 40°C with associated chills, nausea, and dizziness but without diarrhea, vomiting or abdominal pain. There was no history of malaria, leptospirosis, and dengue. On examination, the patient was drowsy and dehydrated and the respiratory system revealed no abnormalities. Blood culture was made using BACTEC (Becton Dickinson, Sparks, MD, USA). The contents of the incubated bottle showed bacterial growth after 12 h. The subcultured colonies uniformly displayed α-hemolysis on the blood agar plates and were oxidase positive and Gram-staining revealed small curved Gram-negative bacilli. API-20E and VITEK2 (bioMérieux) identification confirmed the organism (VC-3) as *V. cholerae*. The tested isolate at NICED was positive for *ompW* and confirmed as serogroup O24. CLSI (2014) guidelines were followed for antimicrobial susceptibility testing. The *V. cholerae* isolate from this case was susceptible to all the antibiotics (**Table 1**). Upon recovery, the patient was discharged after few days of hospitalization.

As shown in **Table 2**, all the isolates of *V. cholerae* identified in this study harbored potential virulence genes encoding for hemolysin (*hlyA*), mannose sensitive hemagglutination (*mshA*), and repeat in toxin (*rtxA*) genes, which might play a role in the disease process including cytotoxicity and invasion. However, the other genes encoding for CT, Stn, Chx and structural genes of T3SS including VcsJ2, VspD, VcsVUQ2, and VcsRTCNSZ were not detected in the PCR assay (**Table 2**). Clinicians should consider *V. cholerae* non-O1/non-O139 as one of the potential etiologic agents in cases of invasive infections. Diagnosis of *V. cholerae* non-O1/non-O139 from extraintestinal infections can be a challenging because of its uncommon description in the literature. Most microbiology laboratories in India diagnose *V. cholerae* infection from suspected cases of cholera or cholera-like diarrhea. Diagnosis of these serogroups can be confirmed by biochemical identification and agglutination with specific antisera. However, this process consumes time and there are no recommended standard methods in the conventional identification.

**Table 2:** Phenotype and virulence gene profiling of *V. cholerae* non-O1/non-O139 isolates.

Isolate	Source	Serogroup	Gene-specific PCR assay results						
			*ompW*	*rtxA*	*mshA*	*hlyA*	T3SS structure genes	*stn*	*ctxAB*	*chxA*
U13533	Urine	O2	+	+	+	+	-	-	-	-
B748	Blood	O8	+	+	+	+	-	-	-	-
SRMC-1	Blood	O10	+	+	+	+	-	-	-	-
VC-3	Blood	O24	+	+	+	+	-	-	-	-

## Discussion

*V. cholerae* non-O1/non-O139 is ubiquitously present in the aquatic environments. Infections caused by these vibrios are often associated with ingestion of contaminated seafood/exposure to coastal waters. The most common symptoms of infection caused by this pathogen are mild to moderate gastroenteritis or wound infections (Lan et al., 2014). These vibrios were also reported to cause biliary tract infection, primary septicemia, peritonitis, skin and soft tissue infections, UTI and pneumonia (Sack et al., 2004; Sangaré et al., 2006; Dutta et al., 2013). In this study, we found different clinical manifestations caused by *V. cholerae* non-O1/non-O139 with diverse serogroups.

*V. cholerae* non-O1/non-O139 are increasingly recognized as a potential etiologic agent in cases of invasive infections (CDC, 2014; Chen et al., 2015; Hao et al., 2015). Timely detection of this pathogen is important in the effective clinical management. Currently, microbiology laboratories in India diagnose *V. cholerae* infection mostly by biochemical and serological testes. However, antisera specific for the non-O1/non-O139 *V. cholerae* serogroups are available only in the reference laboratories. Considering this, the suspected isolates can be tested for *ompW* gene that is highly conserved in *V. cholerae* (Nandi et al., 2000).

In all the four cases, there was no history of consumption of seafood or exposure to seawater and hence, the exact nature of the exposure of patients to *V. cholerae* remains unknown. Antibiotics are essential for the proper management of extraintestinal infections caused by *V. cholerae*. Currently, *V. cholerae* non-O1/non-O139 isolates from many locations are susceptible to most of the antibiotics like β-lactams, fluoroquinolones, trimethoprime-sulfamethoxazole, tetracycline, and chloramphenicol (Sangaré et al., 2006). All the isolates encountered in this study were susceptible to most of the antibiotics prescribed in the treatment of extraintestinal infections and thus the practice can be continued in the treatment of *V. cholerae* non-O1/non-O139-mediated infections.

It is well-known that immunocompetent individuals are vulnerable to several bacterial, viral and parasitic infections. Although the exact mechanism of pathogenesis of extraintestinal infection caused by *V. cholerae* non-O1/non-O139 is not clearly understood, involvements of multifactorial virulence factors cannot be ignored (Sack et al., 2004; Hao et al., 2015). Though the isolates lacked virulence genes encoding for CT, Stn, Chx and structural genes of T3SS, the potential virulence genes such as *hlyA, mshA*, and *rtxA* were detected in all the isolates. Association of these genes in *V. cholerae* non-O1/non-O139 isolates has been reported in many findings (Ottaviani et al., 2011; Dutta et al., 2013; Khan et al., 2013; Rajpara et al., 2013). Since *V. cholerae* non-O1/non-O139 is endemic in many regions, there should be a standby schema in place for routine screening this organism from septicemia/UTI cases. This will help clinicians to make a timely intervention and appropriate management of cases in hospital settings.

## Conclusion

Generally, *V. cholerae* belonging to non-O1/non-O139 serogroups have been considered as an opportunistic pathogen, which occasionally causes severe disease. Considering increasing number of reports, appropriate testing and timely interventions are essential.

## Author Contributions

GC, SS, and TR confirmed, did all the PCR assay and serotyped all the strains; SJ investigated case 1; SB and AB investigated case 2; US investigated case 3 and BB investigated case 4. TR wrote and edited the manuscript.

## Conflict of Interest Statement

The authors declare that the research was conducted in the absence of any commercial or financial relationships that could be construed as a potential conflict of interest.
